# Biochemical characterization of collagen I in Warmblood Fragile Foal Syndrome horse lysyl hydroxylase 1 mutation.

**DOI:** 10.17912/micropub.biology.001399

**Published:** 2025-01-03

**Authors:** Yoshihiro Ishikawa, Sara F. Tufa, Douglas R. Keene, Hans Peter Bächinger, Nena J Winand

**Affiliations:** 1 Department of Ophthalmology, University of California, San Francisco, San Francisco, California, United States; 2 Micro-Imaging Center, Shriners Children's, Portland, Oregon, United States; 3 Department of Biochemistry and Molecular Biology, Oregon Health & Science University, Portland, Oregon, United States; 4 Department of Molecular Medicine, College of Veterinary Medicine, Cornell University, Ithaca, New York, United States

## Abstract

Mutations in the collagen-modifying enzyme lysyl hydroxylase 1 (LH1) cause Warmblood Fragile Foal Syndrome (WFFS) in horses. We investigated the impact of this mutation on collagen structure and function. Our results show that LH1 deficiency leads to reduced lysine hydroxylation, altered collagen fibril organization, and tissue abnormalities resembling human Ehlers-Danlos syndrome. These findings highlight the critical role of LH1 in collagen biosynthesis and provide insights into the pathogenesis of WFFS.

**Figure 1. Characterization of LH1 mutant tissues and collagen I:**
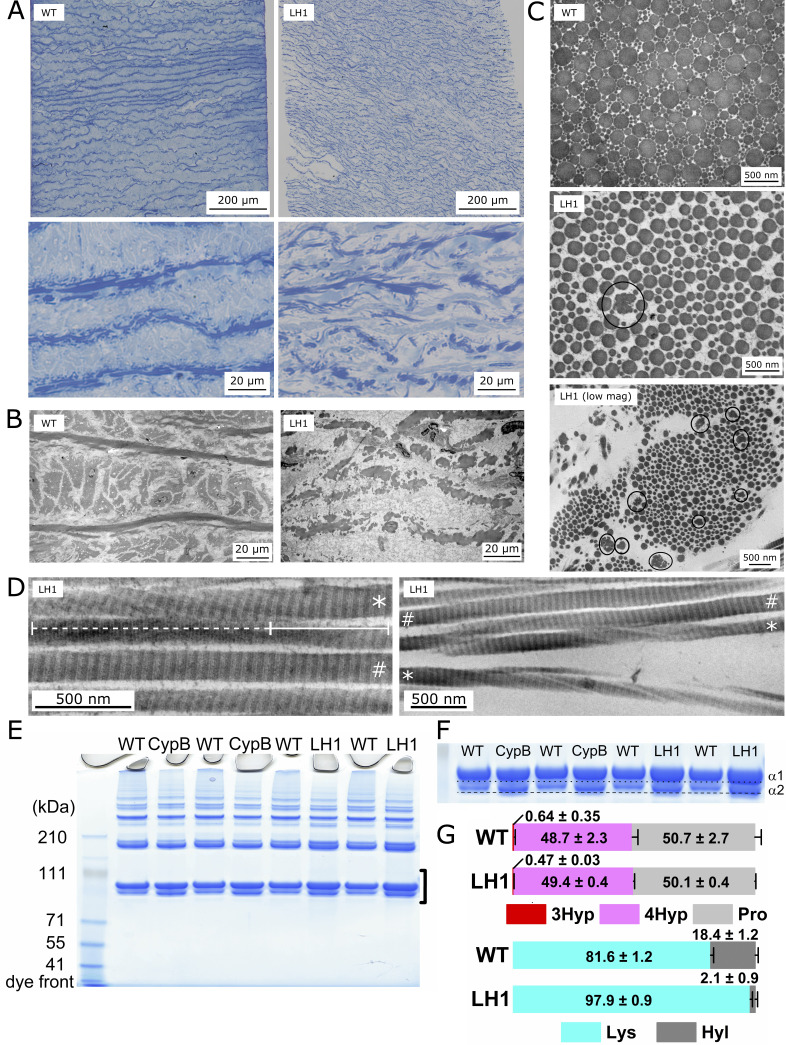
( ***A)*** Light microscopy (LM) demonstrates fragmentation of the elastic lamina of the LH1 throughout the LH1 aorta compared to WT (upper panels of ***A*** ). At higher magnification, both LM (bottom panels of ***A*** ). ( ***B*** ) Transmission electron microscope (TEM) demonstrate fragmentation of every elastin fiber within the LH1 mutant aorta compared to intact elastin fibers within the WT aorta. ( ***C*** ) TEM demonstrates a looser organization of fibrils and occasional “cauliflower” fiber profiles (○) within the LH1 tendon as compared to WT tendon. Differences in fibril diameter may be due to the age difference of LH1 (juvenile) and WT (5yr) tissues. ( ***D*** ) Two representative TEM images of LH1 mutant skin demonstrate both normal (#) and also twisted (*) collagen fibrils. The solid line indicates an aligned fibril; the dashed line indicates a twisted region. ( ***E*** ) SDS-PAGE analysis of purified pepsin treated skin collagen I isolated from LH1 and CypB mutant horse and WT control. Each sample in the SDS-PAGE gel represents a biological replicate, i.e. an independently prepared collagen sample from the tissue. ( ***F*** ) Magnified image of the bracket area in ( ***E*** ). The dotted and dashed line in the gel images indicate the front of the protein bands of the α1 and α2 chain of collagen I, respectively. ( ***G*** ) The ratio of post-translational modifications in proline (3Hyp + 4Hyp + Pro = 100) and lysine (Lys + Hyl = 100) in skin WT and LH1 mutant collagen I are demonstrated as bar graphs. The values of amino acids were obtained using amino acid analysis. Values are given as means ± S.D. Biological replicates were n = 3 for each genotype. [3Hyp(red); 3-hydroxyproline, 4Hyp (pink); 4-hydroxyproline n, Pro (light gray); unmodified proline, Hyl (blue); hydroxylysine, Lys (Dark gray); unmodified lysine].

## Description

The collagen superfamily is one of the most abundant proteins in the animal kingdom (Naba, 2024; Shoulders and Raines, 2009; Tarnutzer et al., 2023) . Mutations in collagens result in connective tissue disorders including Osteogenesis Imperfecta and Ehlers–Danlos syndromes (EDS) (Jovanovic et al., 2022; Lamande and Bateman, 2020; Syx and Malfait, 2024) . In these diseases, not only are there defects in collagens themselves, but mutations in the components of the collagen biosynthetic machinery termed the “molecular ensemble” (Ishikawa and Bachinger, 2013) , that lead to similar pathogenic outcomes in connective tissues (Malfait et al., 2020; Marini et al., 2017) . In horses, mutations in two enzymes of the molecular ensemble have been reported to cause connective tissue disorders: Cyclophilin B (CypB) and Lysyl Hydroxylase 1 (LH1). Mutation in the *PPIB* gene, encoding CypB, the proline isomerase involved in collagen triple helical formation (Ishikawa et al., 2017) , causes Hereditary Equine Regional Dermal Asthenia (HERDA), an autosomal recessive genetic skin disorder that affects Quarter Horses and related breeds (Bowser et al., 2014; Rashmir-Raven and Spier, 2015; Tryon et al., 2007; White et al., 2004) . Mutation in the *PLOD1* gene encoding LH1, the collagen modifying enzyme hydroxylating lysine to hydroxylysine, causes Warmblood Fragile Foal Syndrome (WFFS), an autosomal recessive disorder causing more widespread skin and joint issues than HERDA which primarily affects the skin (Dias et al., 2019; Flanagan et al., 2021; Grillos et al., 2022; Rowe et al., 2021) . WFFS is generally more severe and presents as a congenital disorder affecting foals, while HERDA is progressive and lesions usually become apparent at 1-3 years of age when saddle training begins.

Previous biochemical and structural studies reveal that the CypB mutation impairs collagen biosynthesis, suggesting the potential disease-causing mechanism (Ishikawa et al., 2012) . Mutation in CypB affects its interaction with LH1, resulting in impaired lysine hydroxylation followed by *O* -glycosylations and slower collagen secretion. This change alters collagen fibril structure, leading to a higher proportion of very small fibrils and disorganized fibril alignment. WFFS is also predicted to cause abnormalities in collagen structure, leading to severe pathologies. However, detailed biochemical and structural characterization of collagens in WFFS is lacking. Therefore, we collected tissue samples from a WFFS horse with a known *PLOD1* point mutation (c.2032G > A, p.Gly678Arg) (Monthoux et al., 2015; Winand, 2012) and performed both elastin and collagen ultrastructure analysis using microscopic imaging techniques, and biochemical characterization of collagen I isolated from skins.

Recent studies have indicated that LH1 deficiency is associated with vascular diseases such as aortic aneurysm (Bararu Bojan Bararu et al., 2023; Koenig et al., 2022; Li et al., 2021) . Therefore, we examined the overall structure of the LH1 mutant and wild type (WT) control aorta using light and electron microscopy to investigate how the LH1 mutation affects horse tissue and collagen ultrastructure. We found that elastic fibers were fragmented in the LH1 mutant’s elastic lamina compared to WT using both light and transmission electron microscope (TEM) ( Figure 1A and B). Next, we validated collagen fibril structure using TEM in LH1 mutant tendon and skin. In tendon, cross-section images demonstrated looser fibril packing compared to WT control ( Figure 1C ). Moreover, we also observed occasional disorganized abnormal fibrils (circles in the middle and bottom panels of Figure 1C ), resembling the “cauliflower” deformity of collagen fibrils characteristic of human EDS patient skin (Hausser and Anton-Lamprecht, 1994; Malfait et al., 2010; Pierard et al., 1988) . Most collagen fibrils within control tissues demonstrated a normal “D-period” – the characteristic banding pattern in collagen fibrils (Hodge and Schmitt, 1960) . Also true in LH1 mutant skin, most fibrils demonstrate normal morphology and periodicity (Figure D, hashtag). However, occasional fibrils contained misaligned D-periods ( Figure 1D ; asterisks), appearing as radial twists in the long axis of the fibril. We speculate that this misalignment weakens the structural integrity of the fibril. Note that, as sample collection is challenging in horse studies, we were unable to prepare age-matched WT controls for aorta and tendon (WT control and LH1 mutant are 5-year-old and juvenile, respectively) and to obtain WT controls for skin.

Next, we isolated collagen I from LH1 mutant skin with pepsin digestion followed by sodium chloride precipitation under acetic conditions. Here, we utilized collagen I from WT and mutant CypB horse skin as a reference since mutant CypB collagen I had migrated faster than WT collagen I in an SDS-PAGE gel due to reduced lysine hydroxylation and *O* -glycosylation (Ishikawa et al., 2012) . Collagen I from the LH1 mutant migrated slightly faster than WT on SDS-PAGE gels, which closely resembled the CypB mutant (Figures 1E and F) and implied that post-translational modifications may be impaired. To test this, we performed acid hydrolysis amino acid analysis and found the amount of hydroxylysine to be drastically reduced, while proline modifications remained unchanged ( Figure 1G ). Collectively, these imaging and biochemical analyses suggest that the LH1 mutation affects lysine modification in collagen I, leading to impaired collagen fibril formation and subsequent tissue architectures.

LH1 is a fundamental modifying enzyme in collagen I’s molecular ensemble involving post translational modifications and crosslink formation in the ER and extracellular space (Ellingson et al., 2022; Ishikawa et al., 2021; Takaluoma et al., 2007) . LH1 mutations lead to kyphoscoliotic EDS in humans (Giunta et al., 2005; Rohrbach et al., 2011; Yeowell and Walker, 2000) . Notably, one EDS-causing mutation coincides with this horse LH mutation at the centrally located glycine residue (DYEG ***G*** GCR) of the LH1 carboxyl terminus (Ha et al., 1994) , a sequence conserved in humans, mice, and horses. Therefore, further studies of this mutation 1) are required to clarify whether this mutation leads to the absence of LH1 protein or results in the presence of a non-functional enzyme and 2) could be highly beneficial for understanding the role of LH1 not only in diseases but also in evolutionary and functional aspects. Surprisingly, we observed damage to aortic elastin ( Figure 1A and B) even though elastin lacks hydroxylysines (Schmelzer et al., 2016 ; Yamauchi et al., 2018) , suggesting that defects to elastin are secondary or there are uncharacterized role(s) of LH1 in elastin biosynthesis. In conclusion, the LH1 Gly678Arg mutation that causes WFFS in horses acts by impairing LH1 enzymatic activity, leading to reduced lysyl hydroxylation of collagen I and abnormal collagen fibril structure, as observed in human EDS mutations.

## Methods

***Ethics statement*** – Tissue samples for biochemical analyses were collected from clinically normal horses after euthanasia, and snap frozen in liquid nitrogen. Tissues for light and electron microscopy imaging were similarly collected and fixed as described (Animal Care and Use Protocol 1987-0194, Cornell University).

***LH1 mutant horse material*** – The LH1 mutant foal was diagnosed based on phenotypic criteria by referring veterinarians in consultation with Dr. Winand (Department of Molecular Medicine, College of Veterinary Medicine, Cornell University). The foal was euthanized by the referring veterinarians and tissues were collected and snap frozen for biochemical analyses or fixed as described for light and electron microscopy imaging.

***Light and electron microscopy imaging*** *–* Tissues were fixed in 1.5% glutaraldehyde/1.5% paraformaldehyde (Electron Microscopy Sciences) in Dulbecco’s serum-free media (DMEM) containing 0.05% tannic acid. Samples were rinsed in DMEM then post-fixed in 1% OsO4 overnight, rinsed in DMEM, dehydrated in a graded series of ethanol to 100%, rinsed in propylene oxide, and embedded in Spurr’s epoxy. One-micron sections were stained with an epoxy tissue stain (Electron Microscopy Sciences) and imaged on a Zeiss Imager M2 and post-processed with Zeiss Zen software. Ultrathin sections were cut at 80 nm, contrasted with uranyl acetate and lead citrate, and viewed with an FEI G20 TEM operated at 120 kV with images collected using an AMT 2 x 2K camera.

***Collagen I extraction from LH1 mutant horse skin*** –**** All procedures were performed at 4 ^o^ C. Horse LH1 mutant skin was incubated in excess volume of 0.1 M acetic acid with shaking for several hours. Pepsin was added to a final concentration of 0.25 mg/ml, and tissues were digested overnight. The pepsin treated solutions were centrifuged to remove insoluble material, NaCl was added to a final concentration of 0.7 M to precipitate collagens, and the solution was incubated overnight. Precipitates were collected by centrifugation at 13,000 rpm for 15 min and resuspended in 0.1 M acetic acid. This solution contained an enriched collagen I and III and was dialyzed in an excess volume of 0.1 M Tris/HCl containing 1.0 M NaCl, pH 7.8, and NaCl was then added to a final concentration of 1.8 M to remove type III collagen. This solution was centrifuged at 13,000 rpm for 30 min, and additional NaCl was added to a final concentration of 2.4 M to the supernatant. After incubating overnight, the solution was centrifuged at 13,000 rpm for 30 min. The pellets containing skin collagen I were resuspended in 0.1 M acetic acid and dialyzed against 0.1 M acetic acid to remove remaining NaCl.

***Collagen I from WT and CypB mutant horse skin*** – Normal (WT) and CypB mutant horse skins were collected after euthanasia, and snap frozen in liquid nitrogen (Animal Care and Use Protocol 1987-0194, Cornell University) (Ishikawa et al., 2012) . Collagen I from these skins were isolated using the same procedure described above.

***SDS-gel analyses*** – Purified collagen I samples were run on a 3-8 % Tris/Acetate gel (Invitrogen) in the presence or absence of DTT with boiling denaturation. Gels were stained with GelCode Blue Stain Reagent (Thermo Scientific).

***Amino acid analysis*** – Acid hydrolysis was performed in 6 x 50-mm Pyrex culture tubes placed in Pico Tag reaction vessels fitted with a sealable cap (Eldex Laboratories, Inc., Napa, CA). Samples were placed in culture tubes, dried in a SpeedVac (GMI, Inc. Albertville, MN), and then placed into a reaction vessel that contained 250 ml of 6 M HCl (Pierce) containing 2% phenol (Sigma-Aldrich). The vessel was then purged with argon gas and evacuated using the automated evacuation workstation Eldex hydrolysis/derivatization workstation (Eldex Laboratories, Inc.). Closing the valve on the Pico Tag cap maintained the vacuum during hydrolysis at 105 °C for 24 h. The hydrolyzed samples were then dried in a Savant SpeedVac. The dried samples were dissolved in 100 ml of 0.02 M HCl containing an internal standard (100 µM norvaline; Sigma). The analysis was performed by ion-exchange chromatography with post-column ninhydrin derivatization and visible detection (440 nm/570 nm) with a Hitachi L-8800A amino acid analyzer (Hitachi High Technologies America, Inc., San Jose, CA) running the EZChrom Elite software (Scientific Software, Inc., Pleasanton, CA). Three technical replicates were performed in each analysis.
